# Focused ultrasound blood-brain barrier opening reveals a paradoxical remote metabolic response in the primate brain

**DOI:** 10.1126/sciadv.aed4944

**Published:** 2026-07-17

**Authors:** Soroosh Sanatkhani, Dong Liu, Fabian Munoz, Jack Grinband, Elisa E. Konofagou, Vincent P. Ferrera

**Affiliations:** ^1^ Zuckerman Mind Brain Behavior Institute, Columbia University, New York, NY, USA.; ^2^ Department of Psychiatry, Columbia University, New York, NY, USA.; ^3^ Department of Radiology, Columbia University, New York, NY, USA.; ^4^ Department of Biomedical Engineering, Columbia University, New York, NY, USA.; ^5^ Department of Neuroscience, Columbia University, New York, NY, USA.

## Abstract

Low-intensity focused ultrasound (LIFU) is a promising technique for opening the blood-brain barrier (BBB) for drug delivery, but its physiological consequences in remote brain regions remain a major blind spot for clinical safety and efficacy. To address this gap, we performed the quantitative mapping of brain metabolism following a focal LIFU-induced BBB opening in a nonhuman primate model, using quantitative BOLD MRI to measure the oxygen extraction fraction (OEF). We report a paradoxical response: While the targeted striatum showed no significant metabolic changes, we observed a profound and spatially specific increase in OEF in the homologous contralateral striatum, an effect predominantly driven by the putamen. These findings demonstrate that focal BBB opening is not merely a localized vascular event but a potent neuromodulatory intervention that induces metabolic stress in distant, untreated brain regions, a discovery with critical implications for the safe and effective clinical translation of all focal brain therapies.

## Teaser

Using focused ultrasound to open the BBB paradoxically induces metabolic stress in distant brain regions.

## INTRODUCTION

Low-intensity focused ultrasound (LIFU) is emerging as a technology that can noninvasively deliver mechanical and thermal energy to deep brain structures with unparalleled precision (*1*, *2*). In conjunction with circulating microbubbles (MBs) (*3*–*5*), LIFU transiently and safely opens the blood-brain barrier (BBB), creating a targeted window for the delivery of treatments such as antibodies, gene vectors, or chemotherapeutics. This positions LIFU as a powerful tool for investigating and treating intractable brain disorders like Parkinson’s (*4*, *6*) and Alzheimer’s disease (*7*, *8*), and major depressive disorders (*9*, *10*), where the BBB remains a primary obstacle to effective therapy. However, this picture of focal precision is complicated by the fundamental nature of the brain as a densely interconnected organ. Any focal perturbation within such a system will inevitably have nonlocal consequences. Previous studies have demonstrated that a single application of LIFU for BBB opening (BBBO) alters brain-wide functional connectivity in both rodent (*11*, *12*) and nonhuman primate (NHP) models (*13*). These findings confirm that the intervention induces remote, nonlocal effects. This observation presents a challenge, as the high focal precision of LIFU must be reconciled with the brain’s operation via distributed, interconnected networks.

The current understanding of these remote effects, however, is incomplete. While studies showing altered functional connectivity are important, they may not provide a complete picture of the brain’s physiological response. Functional connectivity, a statistical correlation of blood flow dynamics, is a proxy for neural communication, not a direct measure of tissue health or physiology. A change in connectivity between two regions does not reveal their underlying physiological state; it could reflect a healthy compensatory adjustment or a pathological process. This ambiguity represents a substantial knowledge gap. As LIFU-BBBO advances toward clinical applications, particularly in vulnerable patient populations, a direct physiological assessment is needed to interpret the nature of these remote effects. Without such metabolic data, the full biological impact of the procedure remains uncharacterized, representing a key gap in understanding its broader physiological effects.

To resolve this uncertainty, a direct physiological measurement is needed. The oxygen extraction fraction (OEF), which quantifies the proportion of arterial oxygen extracted by tissue, is a suitable biomarker for this purpose. OEF provides a direct measure of the balance between cerebral oxygen supply, governed by cerebral blood flow (CBF), and the cerebral metabolic rate of oxygen consumption (CMRO_2_) (*14*). Unlike the BOLD (blood oxygenation level-dependent) signal, which reflects multiple contributions, the interpretation of a change in OEF is more physiologically specific. An elevation in OEF, for instance, signals a compensatory response to metabolic stress where oxygen demand outstrips supply (*15*). Conversely, a decrease may indicate luxury perfusion or reduced metabolic activity (*16*). While positron emission tomography has been the gold standard for OEF quantification, its clinical use is limited by high costs and the need for radioactive tracers (*17*). The development of noninvasive magnetic resonance imaging (MRI) techniques such as quantitative BOLD (qBOLD) now permits the robust, high-resolution mapping of OEF (*18*, *19*), making it possible to assess the brain’s physiological reaction to interventions like LIFU-BBBO.

Investigating these remote physiological effects in a translationally relevant model is scientifically necessary, and the complexity of the primate brain makes it an essential model for studying phenomena that are directly relevant to human clinical applications. This study was therefore designed to address the question of whether a focal, transient disruption of the BBB triggers a metabolic response in local or remote brain regions, and to determine the physiological nature of this response. We provide a direct, quantitative measurement of the local and remote metabolic sequelae of LIFU-BBBO in the primate brain. On the basis of the established impact of LIFU on brain-wide functional connectivity (*12*, *13*), we hypothesized that a unilateral BBBO in the striatum would elicit a measurable metabolic response, indexed by a change in OEF, in the untreated, homologous contralateral hemisphere. Understanding this effect is essential for clarifying whether LIFU-BBBO is solely a targeted delivery tool or also a neuromodulatory intervention with widespread physiological consequences, a distinction with important implications for its clinical application.

## RESULTS

**Fig. 1.**
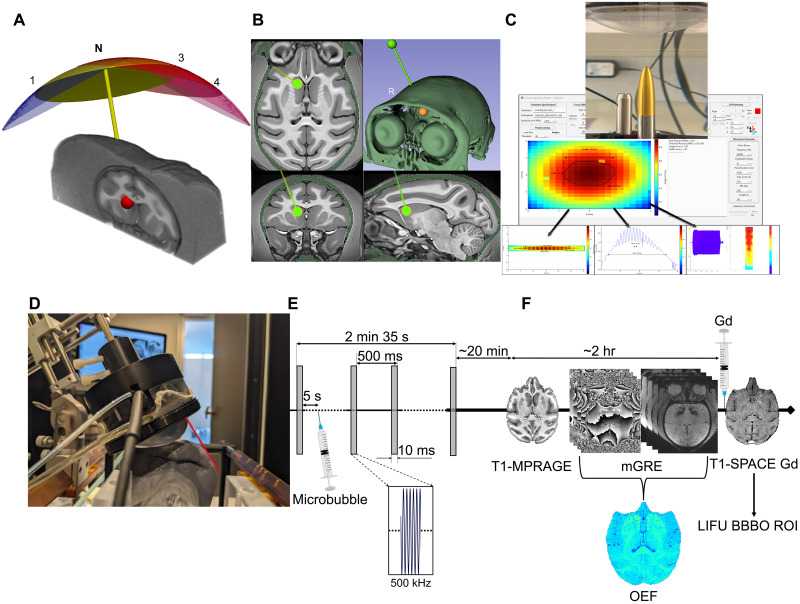
Overview pipeline. (**A**) Acoustic simulations performed in the k-Wave toolbox showing the modeled focused ultrasound transducer and its focus within the brain. (**B**) LIFU treatment planning in 3D Slicer using the subject’s MRI to define stereotaxic coordinates for targeting. (**C**) Acoustic calibration of the LIFU system using CereVista system calibration module. (**D**) In vivo experimental setup showing the transducer coupled to the NHP’s head in a stereotaxic frame (using 3D printed head of the NHP). (**E**) Sonication protocol: 10 ms ultrasound bursts (each containing 5000 cycles at 500 kHz carrier frequency) repeated at 2 Hz for 2 min 35 s (310 total bursts). MB injection occurs 5 s after sonication onset. (**F**) Post–LIFU-BBBO MRI acquisition and data processing pipeline showing study-relevant sequences. MRI acquisitions begin approximately 20 min postsonication. Gadolinium is administered approximately 2 hours (hr) into the MRI session (~2.5 hours postsonication), followed by contrast-enhanced T1-SPACE acquisition.

To systematically investigate the local and remote metabolic consequences of focal LIFU-BBBO, we used a multistage experimental strategy in four NHPs, as summarized in Fig. 1. The study design proceeded from computational validation to in vivo application and analysis. First, acoustic and thermal simulations were performed to establish a safe and effective sonication protocol for targeting the caudate. Following the implementation of this protocol to induce unilateral BBBO, a multimodal MRI protocol was acquired to confirm the location of barrier opening and to generate high-resolution maps of the OEF using the qBOLD model. Last, the resulting OEF changes were quantified using both linear mixed-effects and hierarchical Bayesian models to robustly assess metabolic effects in the targeted and contralateral hemispheres.

### Acoustic and thermal simulation

**Fig. 2.**
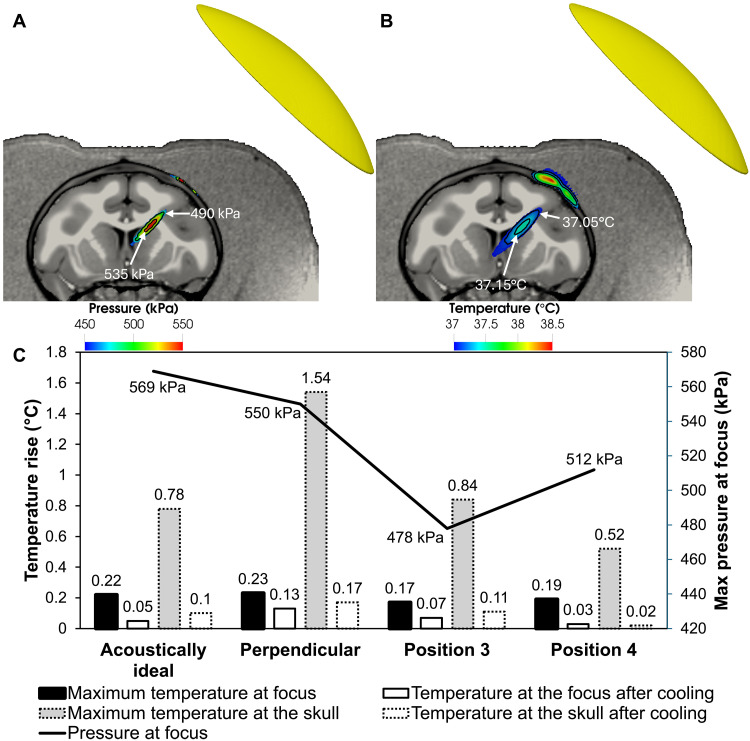
Acoustic simulation results for NHP M1. (**A**) Simulated acoustic pressure map and (**B**) corresponding temperature map overlaid on the subject’s T1-weighted MRI for perpendicular transducer orientation. (**C**) Graph comparing the maximum pressure at the focus (line plot, right *y* axis) with the maximum temperature rise and temperature after 30 s cooling at the focus and skull (bar plots, left *y* axis) across four simulated transducer orientations.

Numerical simulations were performed to optimize the transducer position for targeting the striatum while minimizing heating effects (Fig. 2). Analysis of four different orientations revealed an acoustically ideal path that maximized pressure at the focus (569 kPa) and produced a minimal temperature increase (0.2°C). However, this orientation could not be used because of physical space limitations imposed by the stereotaxic experimental setup. As a result, the perpendicular orientation was selected as the best practical alternative. This approach was confirmed to be both safe and effective, delivering a high focal pressure (550 kPa) with a negligible temperature rise of 0.2°C at the focus and modest heating (1.5°C) at the skull (Fig. 2C). This simulation-guided process validated the chosen methodology for the in vivo experiments.

### T1-SPACE provides superior contrast enhanced image for detection of LIFU-BBBO

**Fig. 3.**
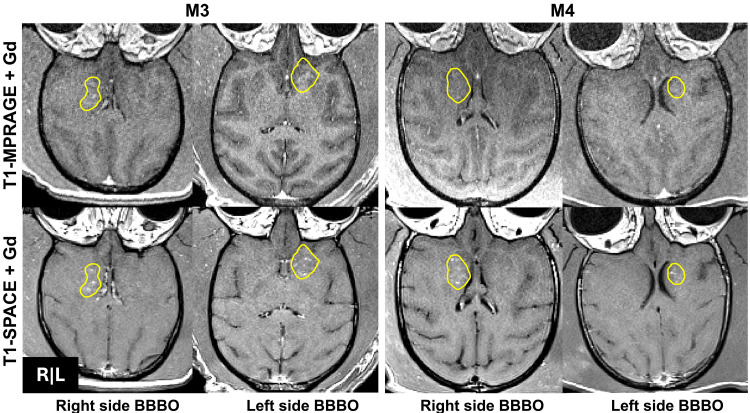
Enhanced BBBO Detection with T1-SPACE sequence. Representative postgadolinium T1-weighted MR images from M3 and M4 comparing the T1-MPRAGE (**top row**) and T1-SPACE (**bottom row**) sequences for detecting BBBO following LIFU + MBs targeting the right/left caudate. Yellow circles indicate regions of gadolinium enhancement, which are clearly visible in T1-SPACE but subtle and difficult to distinguish in T1-MPRAGE.

To confirm LIFU-BBBO, postgadolinium T1-weighted images acquired with magnetization-prepared rapid gradient-echo (MPRAGE) and sampling perfection with application optimized contrast using different flip-angle evolutions (SPACE) sequences were compared. Visual inspection of the images showed that gadolinium enhancement was clearly visible as distinct, hyperintense foci on the T1-SPACE scans. In contrast, on the T1-MPRAGE scans from the same sessions, these areas of enhancement were subtle and often difficult to distinguish from surrounding tissue (Fig. 3). T1-SPACE (*20*) was chosen because of its superior contrast-to-noise ratio and its capability to suppress signals from flowing blood in blood vessels, making it particularly advantageous for detecting BBBO as it selectively visualizes gadolinium that has leaked into brain parenchyma while suppressing signal from residual intravascular gadolinium. The T1-SPACE sequence was therefore used for the delineation of all LIFU-BBBO affected regions of interest (ROIs).

### Focal LIFU-BBBO induces contralateral metabolic response

**Fig. 4.**
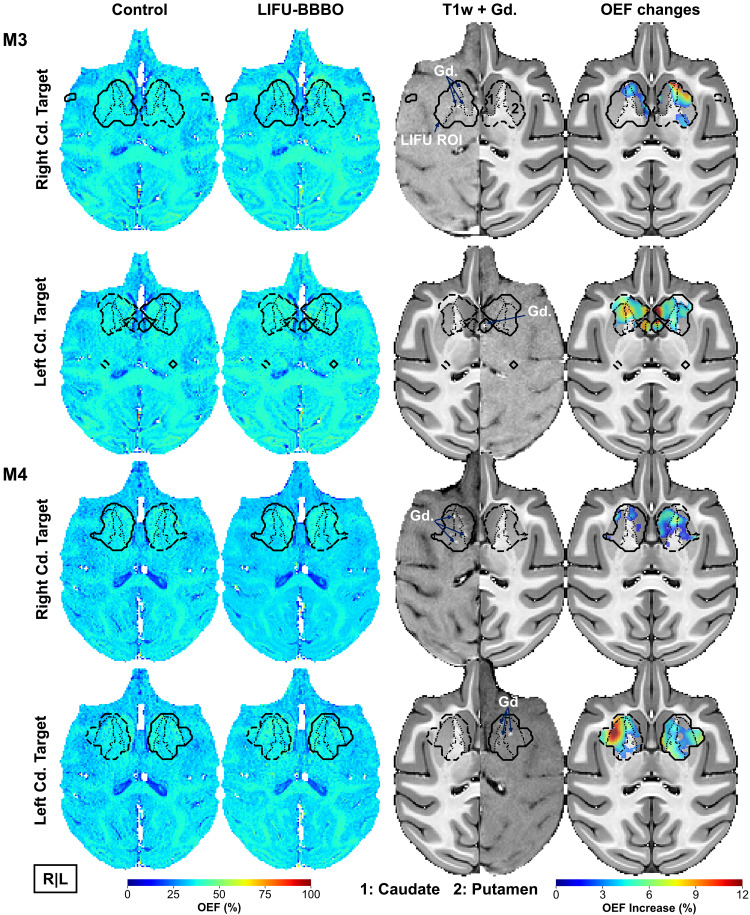
Representative OEF maps and corresponding anatomical images after LIFU-BBBO. Each row displays a different experimental session targeting either the right or left caudate nucleus (Cd. Target). The first two columns show OEF maps for control and LIFU-BBBO conditions, respectively. The third column shows postgadolinium (Gd) T1-weighted images, where dashed lines indicate the LIFU-BBBO ROI and solid lines delineate the caudate (1) and putamen (2). The final column displays the OEF change map, showing an increase in OEF primarily in the homologous contralateral striatum. Representative sessions from M3 and M4 are shown; slices are shown at consistent anatomical positions to facilitate comparison across sessions; note that these may not represent the location of maximum BBBO or peak OEF change for each individual session. Color overlays are thresholded to display only positive OEF changes for visualization clarity; complete unthresholded data including both increases and decreases were used in all statistical analyses. Quantitative volumetric analyses of all sessions are presented in Fig. 5 and Table 1.

Successful and spatially precise BBBO was confirmed in all treatment sessions via gadolinium enhancement on postsonication T1-SPACE MRI. The location of enhancement corresponded directly to the targeted sonication areas in the striatum (Fig. 4, T1w + Gd column). A qualitative inspection of the OEF maps revealed no consistent, large-scale changes at the sonication site itself. Instead, focal increases in OEF were consistently noted in the homologous region of the untreated, contralateral hemisphere (Fig. 4, OEF changes column), prompting a detailed quantitative investigation.

**Fig. 5.**
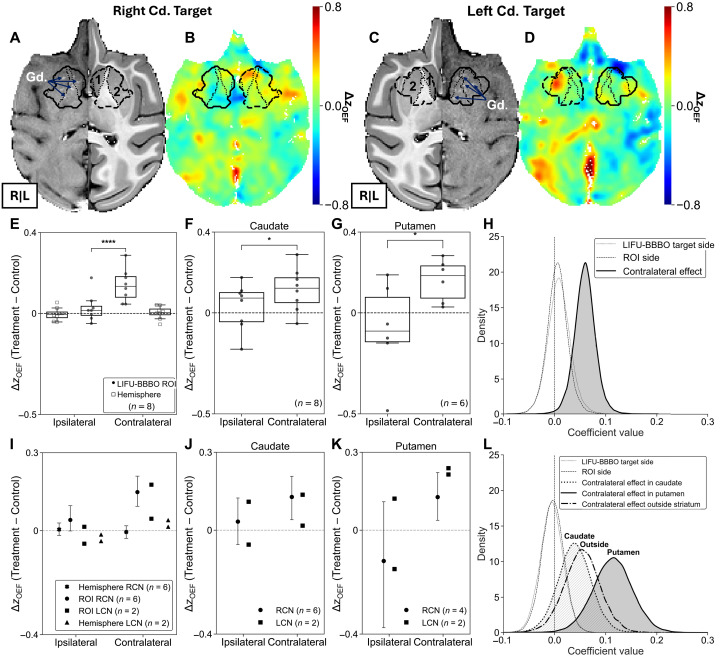
Quantitative analysis of the contralateral metabolic response to LIFU-BBBO. (**A** to **D**) Representative anatomical (T1w + Gd) and OEF change (Δz_OEF_) maps from M4 for sessions targeting the right (A and B) and left (C and D) caudate nucleus. Note the increase in OEF in the homologous contralateral ROI. (**E**) Box plots comparing the change in OEF *z* score between the LIFU-BBBO ROI and the entire hemisphere across all sessions (*n* = 8). A significant increase is observed specifically in the contralateral LIFU-BBBO ROI (*P* < 0.0001, linear mixed-effects model). (**F** and **G**) This contralateral effect is significant when targeting both the (F) caudate (*n* = 8, *P* < 0.05) and (G) putamen (*n* = 6, *P* < 0.05). (**H**) Posterior probability distributions from the primary hierarchical Bayesian model, confirming a credible positive “contralateral effect” (solid gray distribution) whose 95% highest density interval (HDI) does not overlap zero, while main effects for sonication and ROI side are centered near zero. (**I** to **K**) Session-level analysis showing the mean Δ*z*_OEF_ (± 95% CI) for sonications targeting the right (RCN) and left (LCN) caudate nucleus, demonstrating the consistency of the contralateral effect across (I) all ROIs, (J) the caudate, and (K) the putamen. (**L**) Posterior distributions from the sub-regional Bayesian model, indicating the contralateral effect is credibly driven by changes in the putamen.

Quantitative analysis of the change in normalized OEF (Δ*z*_OEF_) confirmed this remote effect. We observed no significant metabolic change in the ipsilateral (treated) ROI, whereas the homologous contralateral ROI showed a robust increase in OEF (Fig. 5, B and D). A linear mixed-effects model found this contralateral effect to be highly significant (*P* < 0.0001). The effect was specific to the sonicated ROI, as no significant changes were observed when averaging across the entire ipsilateral or contralateral hemispheres. This remote metabolic increase was consistently observed whether the target was the caudate nucleus (*n* = 8 sessions) or the putamen (*n* = 6 sessions) and was independent of the hemisphere targeted for sonication.

### Bayesian modeling confirms and quantifies the contralateral effect

To robustly quantify the magnitude of the contralateral increase in OEF while accounting for intersubject variability, we used a hierarchical Bayesian linear regression model. The model confirmed a credible positive contralateral interaction effect, with the mean change in OEF *z*-score being 0.059 [95% highest density intervals (HDI) = (0.017 to 0.099)] (Table 1 and Fig. 5H). In contrast, the main effects for the sonicated side [LIFU-BBBO target side; mean = 0.009 and 95% HDI = (−0.036 to 0.054)] and the ROI hemisphere [ROI side; mean = 0.005 and 95% HDI = (−0.036 to 0.046)] were not credibly different from zero, confirming the specificity of the metabolic change to the homologous contralateral region (Table 1).

**Table 1.** Posterior summary of hierarchical bayesian models. Posterior means, SDs, and 95% highest density intervals (HDI) for model parameters. Upper section: overall contralateral effect within striatal ROI. Lower section: subregional effects by anatomical structure. LIFU-BBBO target side: hemisphere sonicated (right/left). ROI side: hemisphere measured (ipsilateral/contralateral to sonication). Contralateral effect: interaction term testing whether metabolic changes occur consistently in the contralateral ROI versus ipsilateral ROI, independent of which side was targeted. Effects are credible when 95% HDI excludes zero.

LIFU-BBBO effect within ROI	Mean	Standard deviation	HDI 2.5%	HDI 97.5%
Mean baseline	0.076	0.075	−0.073	0.231
σ_Subject-to-subject variability_	0.109	0.099	0.000	0.294
LIFU-BBBO target side	0.009	0.023	−0.036	0.054
ROI side	0.005	0.021	−0.036	0.046
Contralateral effect	0.059	0.021	0.017	0.099
σ_Residual_	0.064	0.019	0.033	0.102
Degrees of freedom	33.971	30.316	0.838	94.151
LIFU-BBBO effect within ROI’s subregions				
Mean baseline	0.071	0.068	−0.067	0.221
σ_Subject-to-subject variability_	0.097	0.101	0.000	0.290
LIFU-BBBO target side	−0.006	0.022	−0.049	0.039
ROI side	−0.004	0.022	−0.047	0.038
Contralateral effect in caudate	0.039	0.033	−0.024	0.104
Contralateral effect in putamen	0.114	0.039	0.038	0.191
Contralateral effect outside striatum	0.054	0.035	−0.015	0.125
σ_Residual_	0.114	0.020	0.076	0.154
Degrees of freedom	25.120	26.158	1.164	78.092

A subregional Bayesian analysis was then performed to identify the anatomical drivers of this remote effect (Table 1 and Fig. 5L). This model demonstrated that the contralateral OEF increase was primarily driven by the putamen, which showed a strong and credible effect (mean = 0.114 and 95% HDI = [0.038 to 0.191]). While positive mean effects were also observed in the contralateral caudate (mean = 0.039) and in regions outside the striatum (mean = 0.054), their 95% HDIs included zero, indicating that these effects were not independently credible (Table 1). Collectively, these results demonstrate that focal LIFU-mediated BBBO elicits a reliable and spatially specific metabolic up-regulation in the untreated, homologous contralateral hemisphere, an effect predominantly driven by changes within the putamen.

To determine if this remote metabolic response is dependent on the magnitude of the targeted intervention, we analyzed an additional subset of sessions where postsonication gadolinium enhancement indicated a subthreshold or absent BBBO. In these sessions, the change in OEF within the contralateral LIFU-BBBO ROI and its caudate subregion exhibited a similar upward trend but failed to reach statistical significance (fig. S2). This suggests that a threshold level of localized BBBO, or the corresponding mechanical acoustic energy delivery, is necessary to trigger the robust network-level metabolic shift observed in the successful treatment sessions.

## DISCUSSION

In the present study, we demonstrate that a focal, unilateral BBBO in the NHP striatum elicits a significant metabolic up-regulation in the homologous contralateral hemisphere, with no corresponding effect at the ipsilateral sonication site. This finding challenges the view of LIFU-BBBO as a purely localized procedure and repositions it as a neuromodulatory intervention with widespread physiological consequences. By quantifying this remote metabolic response using qBOLD MRI to map the OEF, this study addresses a critical gap in understanding the full physiological impact of this emerging therapeutic technology. The key contributions of the present study are as follows: (i) The first quantitative evidence of a remote metabolic response to focal LIFU-BBBO in the primate brain; (ii) the characterization of this response as a paradoxical increase in OEF, which is spatially specific to the contralateral hemisphere and absent at the local sonication site; and (iii) the identification of an anatomical specificity to this remote effect, which our analysis reveals is predominantly driven by the putamen.

The OEF is a fundamental biomarker of brain tissue health, quantifying the proportion of oxygen extracted from the arterial blood supply to meet the brain’s high metabolic needs. According to the Fick principle, the CMRO_2_ (*21*, *22*) is directly proportional to both CBF and OEF (${\text{CMRO}}_{2}\propto \text{CBF}\times \text{OEF}$). OEF serves as a direct and sensitive index of the brain’s metabolic state. An increase in OEF thus signifies a shift in this metabolic balance where local oxygen demand (CMRO_2_) exceeds the available oxygen supply delivered by CBF. In this study, OEF was quantified noninvasively using the qBOLD MRI technique, which estimates OEF by modeling the transverse signal relaxation (T2^∗^) caused by the paramagnetic properties of deoxyhemoglobin in the venous vasculature (*22*–*25*).

Before interpreting the mechanistic implications of this metabolic response, we address its magnitude and spatial consistency. The observed absolute OEF elevation (~2 percentage points) is physiologically meaningful when contextualized against established thresholds for metabolic compromise. Uchida *et al.* (*26*) defined penumbral tissue at risk as OEF elevations exceeding 51.5%, representing approximately 6 to 7 percentage points above normal baseline. Even modest OEF elevations predict future stroke risk in patients with arterial occlusive disease (*16*, *27*, *28*). Our observed elevation is thus subischemic but represents a measurable shift toward metabolic stress. The statistical robustness is supported by convergence across two independent analytical frameworks (linear mixed-effects and hierarchical Bayesian models; Fig. 5 and Table 1), spatial specificity to the contralateral ROI, and replication across sessions despite spatial variability in LIFU-BBBO targeting. When all eight session ROIs are intersected, no single voxel was targeted in every session, yet the contralateral effect remained significant in both session-by-session and averaged analyses (fig. S1 and table S1). This demonstrates that the metabolic response is not an artifact of anatomical targeting but rather a network-level phenomenon triggered reliably when any portion of the ipsilateral striatum undergoes focal LIFU-BBBO. Whether this network-level modulation generalizes to other brain targets remains to be investigated.

The observed elevation in OEF in the contralateral hemisphere therefore signifies a shift in this metabolic balance. This finding indicates that the focal ipsilateral BBBO induces a state of heightened metabolic demand relative to perfusion in the unsonicated hemisphere, predominantly within the putamen. This perspective directs the search for an underlying mechanism away from direct effects and toward the principles of interhemispheric communication and network dynamics. The core mechanistic question is whether this remote metabolic shift is driven by a primary, neurally mediated increase in contralateral metabolic demand, or by a primary alteration in hemodynamics, such as a reduction in contralateral blood flow, or a combination of both. The following sections explore several complementary hypotheses to address this question.

### Hypothesis 1—“Steal”: Diversion of CBF from contralateral to ipsilateral striatum

One potential hemodynamic explanation for the contralateral OEF increase is a vascular “steal” phenomenon, where a profound, localized vasodilation at the ipsilateral BBBO site could shunt blood flow away from the contralateral hemisphere. However, this hypothesis is inconsistent with both the known vasculature of the striatum and our experimental findings. The dorsal striatum, including the caudate and putamen, receives its blood supply primarily from the lenticulostriate arteries, which are small branches of the middle cerebral artery (MCA) (*29*). These vessels are widely considered to be end-arteries, characterized by minimal anastomotic connections between their territories (*30*). This angioarchitecture, with few anastomoses between the major penetrating vessels, implies that the functional zones of the striatum have hemodynamically independent vascular beds (*31*). The vascular supplies to the two hemispheres are largely independent, with the main point of communication being the Circle of Willis. This structure is designed to provide collateral supply in cases of major vessel occlusion rather than to facilitate interhemispheric shunting during focal physiological changes. Therefore, the anatomical substrate for a direct, low-resistance pathway that could steal a substantial volume of blood from the contralateral MCA territory to the ipsilateral one is absent.

Most critically, the central premise of this hypothesis, a major hemodynamic event at the sonication site, is not supported by our data. According to hemodynamic theory, a large-scale hyperemia (a profound increase in CBF) without a corresponding proportionate increase in the CMRO_2_ must result in a decrease in OEF (*32*). We observed no credible change in OEF in the ipsilateral ROI, indicating the absence of the kind of extreme hyperemic or metabolic disturbance that would be necessary to initiate such a steal. While LIFU-BBBO can produce a strong local inflammatory response and edema at high MB doses, these effects are mitigated or absent when using optimized, lower-dose parameters (*33*).

### Hypothesis 2—Increased local neural activity in contralateral striatum through corticostriatal projections

The contralateral OEF increase is plausibly explained by elevated local neural activity and CMRO_2_. Under this hypothesis the trigger could be neuromodulatory effects on the ipsilateral frontal cortex, an area incidentally sonicated by the acoustic path to the caudate nucleus. This stimulation can influence the contralateral hemisphere through at least two known pathways in primates. First, a direct, monosynaptic crossed corticostriatal projection originates from intratelencephalic neurons in motor, premotor, and prefrontal cortices, providing a direct route to the contralateral striatum (*34*, *35*). Second, an indirect, polysynaptic pathway exists via the claustrum, which receives extensive cortical input and projects to the striatum, suggesting a role in coordinating cortico-striatal network activity (*36*).

This network-based explanation is strongly supported by the functional-anatomical specificity of our finding, in which a credible OEF increase was observed only in the contralateral putamen. The primate striatum is organized into functionally segregated circuits, with the putamen acting as the primary input station for the motor loop, receiving dense projections from motor and somatosensory cortices (*35*). Therefore, a focal perturbation of the overlying motor and premotor cortex would be expected to channel its remote effects through these homologous motor circuits, resulting in a metabolic change specifically in the contralateral putamen. However, the cortical-striatal projections are predominantly ipsilateral. Hence, this hypothesis is weakened by the absence of increased OEF in the ipsilateral putamen.

### Hypothesis 3—Increased local neural activity in contralateral striatum through activation of corpus callosum

A parallel neural mechanism involves the corpus callosum, which facilitates communication between the two hemispheres (*37*). This interhemispheric dialogue maintains a dynamic equilibrium of brain activity through a balance of excitatory and inhibitory signals (*38*, *39*). Even in a resting or anesthetized state, the hemispheres exert a tonic, mutually inhibitory influence on each other, ensuring a stable baseline of neural activity (*37*, *40*). This interhemispheric inhibition is mediated by transcallosal pathways that are themselves subject to neuromodulation. This hypothesis proposes that the focal LIFU-BBBO acts as a transient disruption to the local neural circuits in the sonicated ipsilateral striatum (*35*, *41*). This local disturbance could impair the ability of the ipsilateral hemisphere to generate its normal tonic inhibitory output. A reduction of this inhibitory signal across the corpus callosum would lead to a state of “transcallosal disinhibition” in the contralateral hemisphere. Released from this constant inhibitory influence, the baseline neural activity in the homologous contralateral circuits would increase (*35*). This neuronal hyperactivity would in turn drive up local metabolic demand (CMRO_2_), providing a direct cause for the observed increase in OEF.

### Hypothesis 4—Reduction of CBF across the entire dorsal striatum

An alternative hemodynamic hypothesis assumes that the contralateral OEF increase is a consequence of a widespread, bilateral reduction in CBF across the dorsal striatum. The sterile inflammatory response known to be triggered by LIFU-BBBO could lead to a bilateral vasoconstriction or hemodynamic suppression (*33*, *42*). This possibility is directly supported by evidence from rodent studies where unilateral LIFU-BBBO in the somatosensory cortex led to a significant reduction in baseline CBF in both the targeted and the contralateral, nontargeted hemispheres (*12*, *43*, *44*).

However, a bilateral reduction in CBF should, in principle, lead to a bilateral increase in OEF to meet metabolic demands. This contradicts our finding of a strictly unilateral OEF elevation. This discrepancy can be resolved if the expected OEF increase on the ipsilateral side is “masked” by the profound local physiological disruption caused by the BBBO procedure itself. It is well-established that LIFU-BBBO significantly attenuates the local neurovascular unit, hindering its ability to respond to hemodynamic demands (*12*). This local disruption could either cause a concurrent physiological suppression of the local CMRO_2_, which would offset the OEF increase, or it could introduce a measurement artifact in the qBOLD signal due to the altered vascular environment, artificially suppressing the measured OEF value. In either scenario, the result would be that the metabolic consequence of the bilateral CBF reduction is only apparent in the unperturbed contralateral hemisphere.

### Hypothesis 5—Change in neurovascular coupling

The observation of an increased OEF presents a physiological paradox. In a healthy system, a neurally driven increase in metabolic demand triggers a robust increase in CBF that typically overcompensates for the oxygen consumption, leading to a decrease in OEF. Our finding of a significant OEF increase strongly implies a state of neurovascular uncoupling, where the CBF response is insufficient to meet the heightened metabolic demand. This interpretation is directly supported by evidence that LIFU-BBBO is a modulator of neurovascular coupling. A key study demonstrated that LIFU-BBBO applied to the rat somatosensory cortex significantly attenuated the local BOLD and CBF responses to a peripheral stimulus, establishing that the procedure itself alters local physiology in a way that hinders its ability to respond to demands for increased blood flow (*43*). Crucially, this attenuation of the vascular response was also observed during a nonneuronal, hypercapnic challenge, indicating that the disruption occurs at the level of the vasculature itself and not just in the upstream neural signaling pathways. While this effect was documented at the sonication site, it raises the possibility of a similar, more subtle, disruption occurring remotely. Such long-range uncoupling could be mediated by circulating inflammatory molecules released from the BBBO site or through neurally mediated pathways that alter the function of the contralateral neurovascular unit.

### Unified hypothesis

We propose a unified hypothesis to explain the contralateral OEF elevation that integrates both primary neural and secondary vascular factors. The initial event is likely a neurally mediated increase in metabolic demand in the contralateral hemisphere. This could be triggered either by the incidental sonication of the ipsilateral frontal cortex, leading to activation of crossed corticostriatal pathways (Hypothesis 2), or by a disruption of the baseline interhemispheric inhibitory balance, leading to transcallosal disinhibition (Hypothesis 3). Both mechanisms would result in neuronal hyperactivity, particularly within the motor-related putamen, consistent with the anatomical specificity of our findings (i.e., increased OEF in putamen). This increased metabolic demand, however, appears to be met with an inadequate blood supply. This supply-demand mismatch is best explained by a concurrent impairment of NVC (Hypothesis 5), where the vasculature in the contralateral hemisphere is unable to mount a sufficient hyperemic response. This concept of FUS-induced NVC disruption is supported by direct experimental evidence. This integrated hypothesis, combining a primary neural trigger with a secondary vascular insufficiency, comprehensively accounts for the remote nature, metabolic signature (increased OEF), and anatomical specificity of our findings.

The discovery that LIFU-BBBO can induce a state of metabolic stress in an untreated brain region carries significant implications for the clinical translation of LIFU-based therapies. This remote effect must be considered when designing clinical trials and assessing safety, particularly in patient populations who may already have compromised cerebrovascular reserve, such as the elderly or individuals with dementia or a history of stroke. This work also underscores that LIFU-BBBO is not merely a drug delivery tool but is a neuromodulatory intervention in its own right, with complex, network-level consequences. This is further supported by studies showing that LIFU can modulate brain-wide waste clearance via the glymphatic system, an effect that has also been observed in the contralateral hemisphere, highlighting the widespread physiological impact of this technology.

The present study has several limitations regarding experimental design and physiological confounds. A key constraint is the experimental design, which involved acquiring baseline and treatment MRI scans on different days. This approach introduces potential variability from session to session, both in the animal’s physiological state and in the precise MRI setup. Furthermore, the use of isoflurane anesthesia is a significant confound, as it is a known vasodilator that can alter baseline CBF, cerebral metabolism, and NVC. Because our qBOLD measurements were acquired at a single time point approximately 2 hours postsonication, the complete temporal dynamics of this remote metabolic response remain uncharacterized. Mapping the exact duration of the OEF elevation was precluded by the physiological risks of subjecting the animals to prolonged continuous anesthesia within a single session, as well as strict ethical limits on the frequency of repeated anesthesia sessions allowed per week. While established safety profiles of this LIFU-BBBO protocol in primates (*45*) suggest that this metabolic stress is transient, future longitudinal studies are required to determine the precise timeline for a return to physiological baseline. In addition, because our experimental design was optimized to detect acute metabolic changes following individual treatments, the current dataset lacks sufficient statistical power to rigorously evaluate cumulative or longitudinal effects across multiple sonication sessions. Understanding the dose-response relationship of repeated exposures therefore remains a critical avenue for future investigation.

A technical limitation involves the acoustic targeting of deep subcortical structures and the resulting heterogeneous morphology of the BBBO. Unlike the uniform focal spots reported in our prior work, this spatial variance arises from the higher acoustic pressures required, high-sensitivity contrast-enhanced MRI, and uncorrected transcranial phase aberrations. Global skull attenuation (~60%) was empirically compensated for using an ex vivo macaque skull and the CereVista calibration module. However, our single-element transducer precludes active phase aberration correction, allowing subject-specific skull topography to distort the ideal acoustic focal ellipsoid (*46*, *47*). While extensive 3D simulations and in vivo validations in the literature demonstrate that multielement phased arrays mitigate this distortion to restore a contiguous focal volume (*48*–*50*), our spatial intersection analysis shows the contralateral metabolic shift is a generalized network response. We therefore hypothesize that a more conformal focal spot would not fundamentally alter these remote neuromodulatory effects, provided the primary striatal node receives adequate acoustic stimulation.

Beyond the technical constraints of acoustic targeting, further limitations pertain to the measurement techniques and the inferential nature of the proposed mechanisms. The qBOLD technique itself provides an indirect estimate of OEF that relies on a biophysical model with several underlying assumptions; as raised in Hypothesis 4, it is possible that the BBBO procedure interferes with the qBOLD signal, complicating the interpretation of the ipsilateral data. Last, because we did not directly measure CBF or neural activity, the proposed mechanisms, including increased CMRO_2_, bilateral CBF reduction, and impaired NVC, remain inferential.

In conclusion, this study provides evidence in a NHP model that focal LIFU-BBBO in the striatum elicits a paradoxical metabolic response in the homologous contralateral hemisphere, characterized by a significant increase in OEF. This discovery indicates that the physiological impact of LIFU-BBBO is not confined to the sonicated target but extends to remote, untreated brain regions, suggesting that the procedure acts as a neuromodulatory intervention in addition to its role as a drug delivery tool. The observation of a remote metabolic stress response has important implications for the clinical translation of LIFU-based therapies. These findings suggest that a comprehensive understanding of the procedure’s biological effects should include the monitoring of remote brain regions, particularly in patient populations with compromised cerebrovascular reserve. Future work should aim to elucidate the precise neural and vascular mechanisms underlying this remote effect to build a more complete model of LIFU’s interaction with the brain.

## MATERIALS AND METHODS

### Animals

A total of four male macaques (M1 to M4), weighing between 7.1 and 14.1 kg, were used in this study. For all procedures, the animals were first sedated with an intramuscular injection of ketamine (10 mg/kg) and dexmedetomidine (0.02 mg/kg) and subsequently anesthetized with 0.7 to 1.1% isoflurane for the duration of the experiment. Throughout the MRI scans, vital signs, including body temperature, electrocardiogram, oxygen saturation, and respiratory CO_2_, were continuously monitored by an Iradimed 3880 MRI-compatible monitoring system (Winter Springs, FL, USA). All experimental procedures involving NHPs were approved by the Institutional Animal Care and Use Committee of Columbia University (animal welfare assurance number: D16-00003).

### LIFU simulation and planning

To guide treatment planning, numerical simulations of transcranial ultrasound propagation were performed for NHP M1 using the k-Wave MATLAB toolbox (*51*). A heterogeneous 3D anatomical model of the head was generated from the animal’s T1-weighted MRI where a computed tomography (CT) scan from a size-matched rhesus macaque was registered to M1’s T1-weighted MRI. This model was segmented into skin, muscle, skull, and brain tissues. Heterogeneous acoustic properties (density and sound speed) were mapped from the CT intensity values, with a porosity-based model of the skull used to account for acoustic aberrations. Other tissue-specific parameters, including acoustic absorption and thermal properties, were assigned from literature values and are detailed in Table 2.

**Table 2.** Acoustic simulation parameters. Parameters were assigned to each tissue compartment (water, skin, muscle, and skull) in the heterogeneous 3D head model.

Acoustic properties		Thermal properties	
Speed of sound	(m/s)	Specific heat	J/(kg·K)
Water	1482.3	Water	4178
Skin	1624	Skin	3391
Muscle	1588.4	Muscle	3421
Skull (min/max)	1855/3360	Skull	1313
Density	**(kg/m^3^)**	**Thermal conductivity**	**W/(m·K)**
Water	998.23	Water	0.6
Skin	1109	Skin	0.37
Muscle	1090	Muscle	0.49
Skull (min/max)	1080/2100	Skull	0.3
Absorption coefficient (α0)	**dB/(cm·MHz)**		
Water	0.0022		
Skin	1.84		
Muscle	0.47		
Skull	2.7		

Acoustic propagation was simulated on a 0.3-mm isotropic grid using the kspaceFirstOrder3D solver. The ultrasound source was modeled as a 500-kHz, 64-mm-diameter, single-element transducer with a 63.2 mm radius of curvature, driven at a source pressure of 50.5 kPa. To identify an optimal acoustic window, simulations were performed for four distinct transducer orientations: one perpendicular to the skull and three angled approaches (Fig. 1A). The resulting 3D acoustic pressure fields were then used to calculate the acoustic energy deposition rate (*Q*), which served as the heat source for thermal modeling via the Pennes bioheat equation. The thermal simulation, implemented with the kWaveDiffusion solver, precisely replicated the experimental pulsed sonication sequence (10-ms bursts at 2 Hz for 2 min) from a baseline temperature of 37°C.

The simulated 3D pressure and temperature maps from each of the four orientations were visualized in ParaView (Kitware Inc.) (*52*) to assess the focal accuracy and predicted temperature rise. This comparative analysis guided the selection of the optimal transducer position, which was then used to create the final LIFU treatment plan in 3D Slicer (*53*) (Fig. 1B).

While the k-Wave solver is extensively validated in the literature for focused ultrasound applications (*54*, *55*), we further confirmed our subject-specific safety margins via an ex vivo thermometry experiment using a macaque skull and an albumin-agar tissue-mimicking phantom. This validation demonstrated excellent agreement with our simulated heating profiles, confirming negligible focal heating and bounding the maximum skull surface temperature (see supplementary text S4).

### LIFU BBBO

A total of eight BBBO sessions were performed across the four NHPs: M1 underwent two sessions (both targeting right caudate), M2 underwent one session (right caudate), M3 underwent three sessions (two right caudate and one left caudate), and M4 underwent two sessions (one right caudate and one left caudate). While all sessions specifically targeted the caudate nucleus, the spatial extent of BBBO (confirmed via gadolinium enhancement) extended to include other regions including the putamen in six of the eight sessions due to anatomical proximity and acoustic beam geometry.

The scalp of each NHP was shaved and depilatory cream was applied to remove all fur for optimal ultrasound coupling. The head was immobilized in a stereotaxic frame (Fig. 1D). A warming blanket was used underneath the NHP throughout the BBBO process to maintain body temperature. A coupling system consisting of an inflated bladder filled with degassed, deionized water was used for acoustic attachment, with conductive gel applied at the interface. The LIFU system consisted of a single-element, 500-kHz transducer (H-107; 63.2 mm radius of curvature, 64 mm outer diameter; Sonic Concepts, Bothell, WA) driven by a function generator (Agilent 33220A; Agilent Technologies, Santa Clara, CA) and a 57 dB radio frequency power amplifier (500S06; E&I, Rochester, NY). The system was calibrated via the CereVista system calibration module (Fig. 1C). The safety of the acoustic parameters and the MB dosing used in this study has been comprehensively evaluated and established in our prior work in NHPs (*45*), which confirmed the absence of microhemorrhage, edema, or structural tissue damage at these specific sonication levels. The caudate nucleus region was targeted on the basis of stereotaxic coordinates (*56*), with a single target sonicated in each session. Sonications consisted of repeated 10-ms ultrasound bursts at a 500 kHz carrier frequency, delivered continuously at a pulse repetition frequency of 2 Hz (2% duty cycle) for 2 min and 35 s, resulting in approximately 310 bursts per sonication session. Five seconds after the start of the sonication, a bolus of in-house manufactured, lipid-shelled (DSPC/DSPE-PEG2000) MBs with a perfluorobutane gas core (4 to 5 μm diameter) was injected intravenously through the saphenous vein at a dose of 2 × 10^8^ MBs/kg (Fig. 1E). A free-field peak-negative pressure of 1.375 MPa was applied, corresponding to an estimated pressure of 550 kPa at the target after an assumed 60% attenuation by the skull. These parameters were chosen to facilitate safe BBBO, as previous studies have shown that tissue damage can be avoided with appropriate ultrasound settings and MB dosage (*45*, *57*).

### MRI acquisition

Approximately 20 min after the LIFU-BBBO procedure, NHPs were transferred to a 3T Siemens Prisma scanner, positioned supine and head-first, and a 15-channel knee coil was used for signal acquisition. The imaging protocol was acquired in the transversal orientation with right-to-left phase encoding and a prescan normalization filter was applied. The protocol began with a T1-MPRAGE sequence [repetition time (TR) = 2700 ms, echo time (TE) = 2.8 ms, inversion time (TI) = 860 ms, flip angle (FA) = 8°, isotropic 0.5 mm resolution, and field of view (FOV) = 128 × 128 × 96 mm^3^], followed by a multiecho gradient echo (mGRE) sequence (TE1 = 3.8 ms, ΔTE = 6.5 ms, 6 to 8 echoes, TR = 41 to 54 ms, FA = 8°, isotropic 0.5 mm resolution, and FOV = 128 × 128 × 96 mm^3^). Last, approximately 2 hours into the MRI session (~2.5 hours postsonication), following an intravenous injection of gadolinium (0.2 ml/kg), a contrast-enhanced T1-SPACE sequence was acquired (TR = 700 ms, TE = 6.4 ms, isotropic 0.5 mm resolution, and FOV = 128 × 128 × 96 mm^3^) (Fig. 1F). In a subset of sessions, postcontrast T1-MPRAGE was also acquired after gadolinium injection to enable direct comparison with T1-SPACE for BBBO detection (Fig. 3).

### Quantitative BOLD modeling

For each subject, the qBOLD analysis was performed on a voxel-wise basis. Initial processing of the mGRE data involved phase unwrapping using the ROMEO algorithm (*58*). The unwrapped complex signal, S(TE), was subsequently modeled using a variation of the qBOLD model (*18*):

$$S\left(\text{TE}\right)=S_{0}\cdot \text{exp}\left(-R_{2}\cdot \text{TE}+i\cdot 2\pi \cdot \Delta f\cdot \text{TE}\right)\cdot F_{\text{BOLD}}\left(\text{TE}\right)\cdot F\left(\text{TE}\right)$$ (1)

In this model, $S_{0}$ is the signal magnitude at TE=0, $R_{2}$ is the transverse relaxation rate, and Δf is the frequency shift from macroscopic field sources. The contribution from these macroscopic field inhomogeneities, F(TE), was corrected using the voxel spread function method (*59*). The BOLD-specific signal attenuation, $F_{\text{BOLD}}\left(\text{TE}\right)$, which arises from deoxygenated blood in the veins and adjacent microvasculature, was modeled as

$$F_{\text{BOLD}}\left(\text{TE}\right)=1-\frac{\xi }{1-\xi }f_{s}\left(\delta \omega \cdot \text{TE}\right)+\frac{1}{1-\xi }f_{s}\left(\xi \cdot \delta \omega \cdot \text{TE}\right)$$ (2)

where ξ represents the deoxygenated cerebral blood volume fraction. The term δω is the characteristic frequency shift at the vessel wall, defined as

$$\delta \omega =\frac{4}{3}\pi \cdot \gamma \cdot B_{0}\cdot \text{Hct}\cdot \Delta \chi_{0}\left(1-Y\right)$$ (3)

For this calculation, the gyromagnetic ratio (γ) was $267.53\times 10^{6}\text{rad}\;s^{-1}T^{-1}$ (*60*), the main field strength ($B_{0}$) was 3 T, hematocrit (Hct) was assumed to be 0.4, and the susceptibility difference between fully oxygenated and deoxygenated blood ($\Delta \chi_{0}$) was 0.27 parts per million (*61*). The key variable, Y, is the blood oxygenation level. The function $f_{s}$, which represents the signal decay from the vessel network, is defined by the generalized hypergeometric function, F21:

$$f_{s}\left(\delta \omega \cdot \text{TE}\right){=}_{1}F_{2}\left([-\frac{1}{2}];[\frac{3}{4},\frac{5}{4}];-\frac{9}{16}{\left(\delta \omega \cdot \text{TE}\right)}^{2}\right)-1$$ (4)

The four key biophysical parameters, $S_{0}$, $R_{2}$, ξ, and Y, were estimated for each voxel by fitting Eq. 1 model to the complex mGRE data. The fitting procedure was implemented in Python, using the Adam optimizer within the PyTorch library for GPU-accelerated computation and the NumPy library for numerical data handling. Details of optimizer configuration, parameter initialization and constraints, fit quality assessment, and a sensitivity analysis of the assumed model parameters (Hct and $\chi_{0}$) are provided in the supplementary text S3. Last, the OEF map was calculated directly from the estimated blood oxygenation level as OEF=100×(1−Y)%.

### MRI data preparation

Image processing was conducted using custom Python scripts that incorporated tools from AFNI (Analysis of Functional NeuroImages) (*62*). For each session, the T1-MPRAGE image was coregistered to the corresponding mGRE volume. All data were then registered to the NIMH Macaque Template v2 (NMT2) (*63*) atlas using AFNI’s animal_warper tool (*64*). The resulting transformation matrices were saved for subsequent use, and the brain masks generated by this process were manually refined with ITK-SNAP (*65*).

Quantitative OEF maps were generated from the mGRE data as described previously and were warped into NMT2 space using the saved transformations. In the NMT2 space, an ROI representing the LIFU-BBBO-affected area was manually drawn. This was guided by the visible contrast enhancement on the registered, gadolinium-enhanced T1-weighted images. To ensure full coverage of the affected tissue, this ROI was then dilated and expanded along the known trajectory of the ultrasound beam. A corresponding contralateral ROI was then created by mirroring this region across the midline.

### Statistical analysis

All statistical analyses were conducted using custom scripts in Python. To control for intersession variability, OEF maps were first converted to voxel-wise *z* scores relative to each volume’s whole-brain mean and standard deviation. The primary outcome metric, the change in mean OEF *z* score ($\Delta {\bar{z}}_{\text{OEF}}$) between treatment and averaged baseline control sessions, was assessed within each ROI. These effects were analyzed using a linear mixed-effects model with the following fixed effects: (i) LIFU-BBBO target side (which hemisphere was sonicated: right versus left), (ii) ROI side (which hemisphere the ROI measurement was taken from: ipsilateral versus contralateral to the sonicated hemisphere), and (iii) their interaction term (the “contralateral effect”). The contralateral effect interaction term specifically tests whether there is a consistent metabolic response in the contralateral ROI compared to the ipsilateral ROI, regardless of which hemisphere was sonicated. A random intercept for each subject was included to account for repeated measures; a *P* value <0.05 was considered significant. To further quantify the magnitude of these effects, a complementary hierarchical Bayesian linear regression was implemented [PyMC (*66*)]. This model also included subject-specific random intercepts and an interaction term to isolate the homologous contralateral effect, but used a robust Student’s *t* distribution for the likelihood. For this analysis, an effect was deemed statistically credible if the 95% HDI of its posterior distribution did not overlap zero.
