# Supplementary material for: Focused ultrasound blood-brain barrier opening reveals a paradoxical remote metabolic response in the primate brain
Source: Sci Adv. 2026 Jul 17;12(29):eaed4944. doi: 10.1126/sciadv.aed4944 (PMC13378551; doi:10.1126/sciadv.aed4944)
Supplement: Supplementary file 1 — Supplementary Text S1 to S4 Figs. S1 to S3 Table S1 [file sciadv.aed4944_sm.pdf]

Science Advances
AAAS

Supplementary Materials for

Focused ultrasound blood-brain barrier opening reveals a paradoxical remote metabolic response in the primate brain

Soroosh Sanatkhani *et al.*

Corresponding author: Soroosh Sanatkhani, ss6481@columbia.edu; sorooshsanatkhani@outlook.com

Sci. Adv. **12**, eaed4944 (2026)
DOI: 10.1126/sciadv.aed4944

This PDF file includes:

Supplementary Text S1 to S4
Figs. S1 to S3
Table S1

Supplementary Materials

S1. Robustness of Group-Level OEF Analysis to Cross-Subject Averaging Methods

To assess the robustness of the group-level OEF analysis to methodological choices, we performed three variants of the cross-subject averaging pipeline (**Fig. S1**). Simple voxel-wise averaging across sessions is inherently limited by significant inter-session variabilities. These include differences in the precise spatial extent of the targeted BBBO, technical MRI setup inconsistencies across acquisition days, and variations in the physiological status or anesthetic depth of the subjects. Despite these confounding factors, which systematically attenuate spatially focal signals, all variants averaged raw OEF volumes across sessions (with left caudate LIFU-BBBO sessions mirrored to the right orientation) before applying within-brain z-score normalization. The variants differed in (i) subjects included and (ii) FUS ROI definition. Variant A included all four subjects (M1 to M4) with all available treatment sessions and used the union of LIFU-BBBO ROI masks across sessions. For the intersection-based approach, we initially attempted to compute the voxel-wise intersection of LIFU-BBBO ROI masks across all four subjects; however, this yielded zero overlapping voxels, reflecting the inter-subject variability in LIFU-BBBO targeting geometry. We therefore restricted the intersection and union analyses to M3 and M4, whose LIFU-BBBO ROIs exhibited the greatest spatial overlap. Variant B included only M3 and M4 (each with one right and one left caudate LIFU-BBBO session) and used the intersection of their LIFU-BBBO ROI masks, yielding a smaller ROI (2,111 ipsilateral voxels vs. 18,416 in Variant A). Variant C used the same two subjects as Variant B but with the union of LIFU-BBBO ROI masks. Mann-Whitney U tests comparing voxel-wise Δz_{OEF} distributions between the ipsilateral (treated) and contralateral (mirrored) LIFU-BBBO ROI consistently showed significant lateralized differences across all three variants (**Table S1**). The difference was significant in the putamen sub-region across all variants, while the caudate sub-region reached significance in Variant C but not in Variants A or B. Overall, these sensitivity analyses confirm that the lateralized OEF increase within the FUS-targeted region is a consistent finding that does not depend on the specific choice of subject pool, number of treatment sessions, or ROI combination strategy (union vs. intersection).

S2. Metabolic Response in Sub-Threshold LIFU-BBBO Sessions

To investigate whether the observed remote metabolic response is associated with the magnitude of the localized blood-brain barrier opening, we analyzed a separate subset of treatment sessions characterized by sub-threshold or absent BBBO. These sessions were identified qualitatively by a lack of substantial gadolinium enhancement on post-sonication T1 and were excluded from the primary efficacy analysis. Voxel-wise z-score normalization and region-of-interest extractions were performed identically to the successful treatment sessions. While the normalized OEF Δz_{OEF} within the contralateral LIFU-BBBO ROI and its isolated caudate subregion showed a positive trend, the effect did not reach statistical significance in the absence of a robust focal barrier disruption (**Fig. S2**).

S3. Quantitative BOLD Model Fitting

All computations were performed in double precision. The generalized hypergeometric series (**Equation 4**) was evaluated using precomputed coefficients up to 40 terms with an early termination tolerance of 10^{-6}. The Adam optimizer was configured with a learning rate of 0.01, default momentum parameters ($\beta_1 = 0.9$, $\beta_2 = 0.999$), and run for up to 500 iterations with early stopping triggered when the loss fell below 10^{-3}. The loss function was defined as the mean squared difference between predicted and measured signal magnitudes, normalized by the mean squared magnitude across all masked voxels, making the optimization invariant to absolute signal scaling. Parameters were initialized as $S_0 = 1.1\times|S(TE_1)|$ (computed per voxel from the first echo), $T_2 = 60$ ms, $\zeta = 0.3$, and $Y = 0.5$. To maintain physiological plausibility, parameters were

clamped after each gradient step to the following ranges: $S_0 \in [0.01,5000]$, $T_2 \in [30,2500]$ ms, $\zeta \in [0.001,0.99]$, and $Y \in [0.1,0.9]$. The brain mask was eroded by one voxel prior to fitting to mitigate edge effects.

Per-voxel fit quality was assessed by computing the mean squared error between the fitted model and the measured signal across echo times. Voxels with MSE exceeding 1000 were excluded from the final parameter maps, as high residual error indicates poor model convergence or violation of model assumptions such as partial voluming with CSF or large vessels.

The qBOLD model relies on two physiological constants that are not directly measured: hematocrit ($Hct = 0.4$) and the susceptibility difference between fully oxygenated and deoxygenated blood ($\Delta\chi_0$=0.27 ppm). These parameters enter the model exclusively through their product in the frequency shift term $\delta\omega$ (**Equation 3**) and therefore affect estimated OEF in a coupled manner. A ±10% variation in *Hct* (i.e., 0.36 to 0.44, spanning the physiologically plausible range for non-human primates propagates) as a proportional shift in estimated ($1-Y$), corresponding to an absolute OEF change of approximately ±3-4 percentage points at typical cortical values (~30-40%). Similarly, the assumed $\Delta\chi_0$=0.27 ppm is well-established, with reported values ranging from 0.24 to 0.27 ppm (*61*); adopting the lower bound would increase OEF estimates by approximately 4%. Because these constants were held fixed across all subjects and sessions, any systematic bias affects all measurements uniformly and does not influence the within-subject or between-session contrasts reported in this study.

S4. Validation of Thermal Simulations using an Ex Vivo Macaque Skull and Tissue-Mimicking Phantom

To empirically validate the *in silico* thermal predictions and confirm *in vivo* safety margins, an *ex vivo* thermometry experiment was conducted using an excised macaque skull and a tissue-mimicking phantom. The phantom was prepared using an albumin and agar. Temperatures were recorded using an Advanced Energy fluoroptic temperature STB probe (accuracy of ±0.1 ºC) at the calibrated temperature. The probe was positioned sequentially at the inner surface of the skull (the tissue side) and at the acoustic focus. The sonication protocol exactly mirrored the *in vivo* LIFU parameters (without microbubbles), consisting of a 30-second pre-sonication baseline, 120 seconds of sonication, and a 30-second post-sonication cooling period.

As shown in **Fig. S3**, sonication induced a reliable temperature increase at the inner surface of the skull. This observation aligns closely with the maximum skull temperatures predicted by our k-Wave simulations, which reported peak temperatures inside the bone ranging from 0.52 to 1.54 °C depending on transducer orientation. In contrast, no detectable temperature elevation was observed at the acoustic focus. This experimental finding is highly consistent with our thermal simulations, which estimated a focal temperature increase of approximately 0.2 °C. A magnitude of 0.2 °C falls near the absolute accuracy limits of the fluoroptic probe and is thus indistinguishable from baseline thermal noise. Together, these measurements confirm that the low-intensity parameters utilized in this study do not induce meaningful focal heating, and that the simulations accurately bound the maximum expected skull heating *in vivo*.

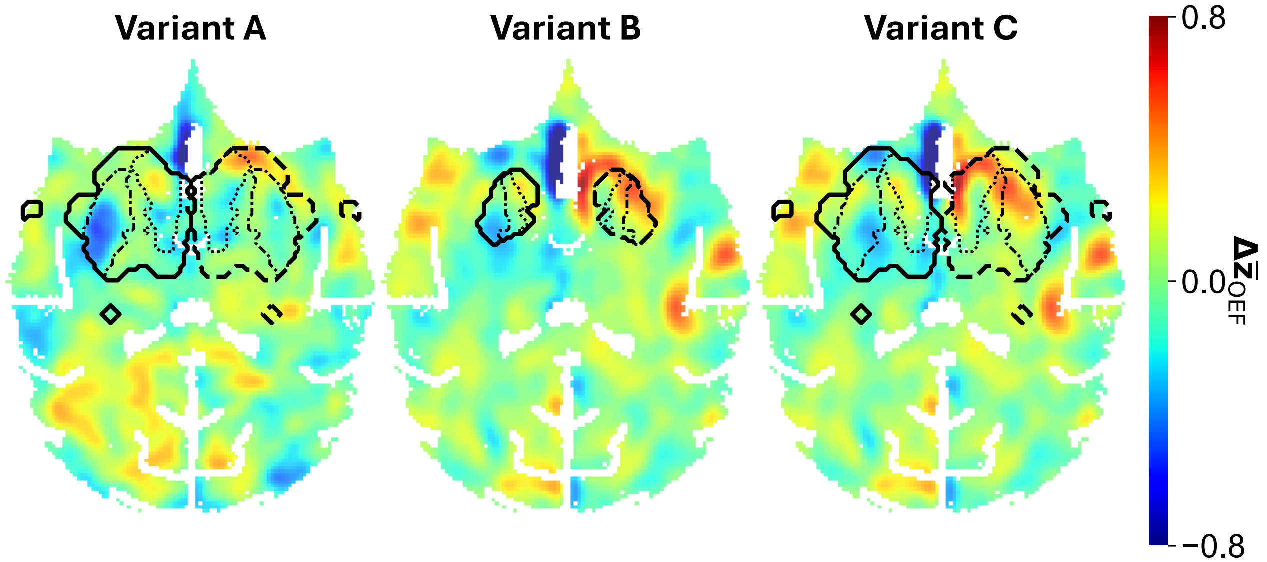

Fig. S1. Voxel-wise average maps of the change in normalized OEF (Δz_{OEF}). *The maps illustrate the spatial distribution of metabolic changes for the three methodological variants (A, B, and C) used to assess the robustness of the contralateral effect. Despite spatial attenuation caused by inter-subject variability in targeting, the contralateral OEF elevation remains visible and statistically significant, specifically when restricting the analysis to sessions with high spatial overlap (Variants B and C)*

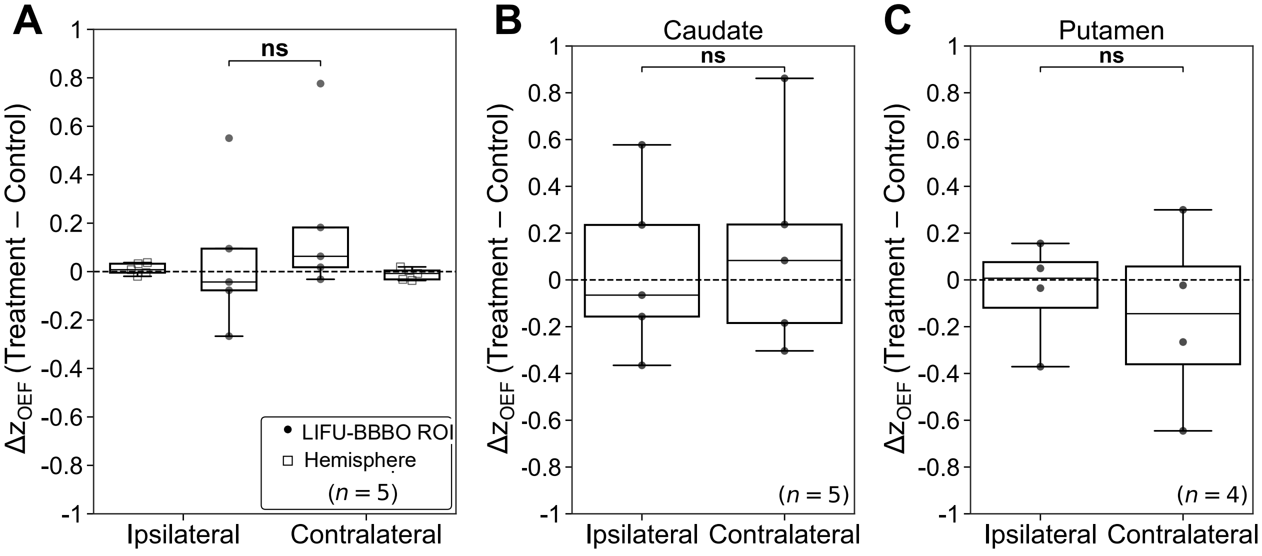

Fig. S2. Quantitative analysis of OEF changes in sub-threshold LIFU-BBBO sessions. Box plots comparing the change in normalized OEF Δz_{OEF} between the ipsilateral (targeted) and contralateral (untreated) hemispheres in sessions where post-sonication MRI revealed sub-threshold or absent gadolinium enhancement. Data are shown for the (**A**) entire LIFU-BBBO ROI, (**B**) the isolated caudate subregion, and (**C**) the isolated putamen subregion. While a slight upward trend in contralateral OEF is visible in the entire ROI and caudate, and a slight downward trend is observed in the putamen, none of the differences between the ipsilateral and contralateral regions reach statistical significance. These null results indicate that a robust, localized BBB disruption is required to elicit a reliable remote metabolic response.

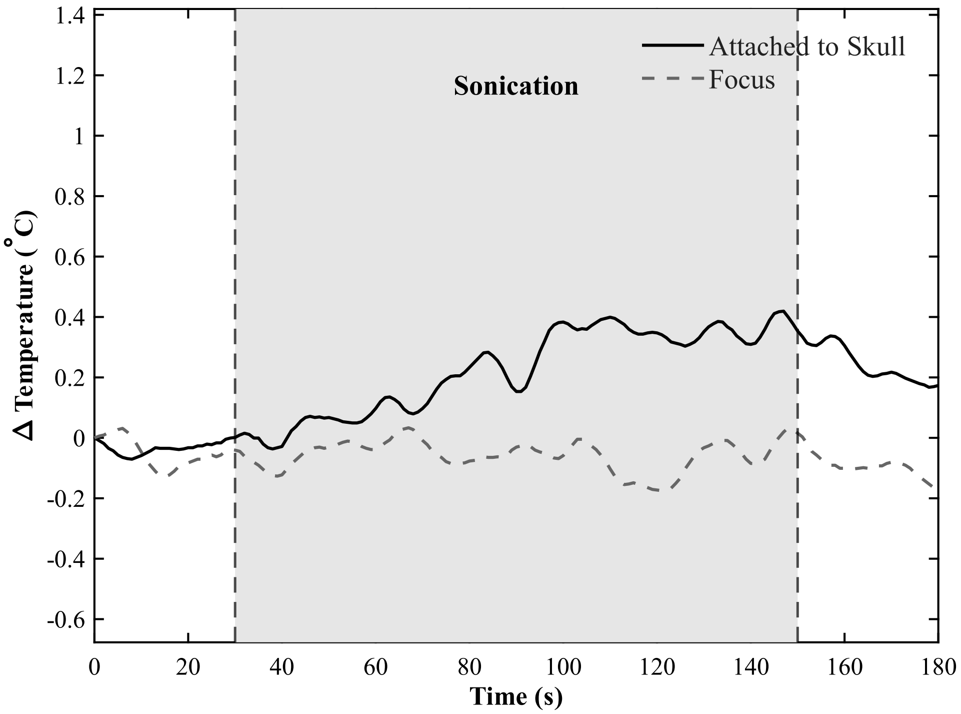

Fig. S3. ***Ex vivo*** **thermometry validation of transcranial low-intensity focused ultrasound.** Temperatures were recorded via fluoroptic probe (Advanced Energy STB) at the inner surface of an excised macaque skull and the acoustic focus within a tissue-mimicking phantom. The shaded region indicates the 120-second sonication. Data were smoothed using a 5-second moving average. The observed temperature elevation at the inner skull surface corroborates *in silico* predictions of maximal heating within the bone. Conversely, no detectable heating occurred at the focus, aligning with the simulated 0.2 °C increase, which borders the probe's accuracy limit (±0.1 °C).

Table. S1. Statistical analysis of averaged OEF changes across methodological variants. *Presents the results of Mann-Whitney U tests comparing voxel-wise* Δz_{OEF} *distributions between the ipsilateral (treated) and contralateral (mirrored) LIFU-BBBO ROIs. Three cross-subject averaging variants were analyzed to assess the robustness of the group-level findings. Variant A includes all four subjects (M1 to M4) and uses the union of LIFU-BBBO ROI masks. Variant B includes only M3 and M4 using the intersection of their ROI masks to account for spatial overlap. Variant C includes M3 and M4 using the union of their ROI masks. Significant lateralized differences were consistently observed, particularly driven by the putamen sub-region.*

Variant A

Region	Side	$\Delta\bar{z}_{OEF}$	σ	p
LIFU-BBBO	Ipsilateral	0.04	0.91	<0.0001
LIFU-BBBO	Contralateral	0.08	0.88	<0.0001
LIFU-BBBO ∩ Caudate	Ipsilateral	-0.08	1.08	0.34
LIFU-BBBO ∩ Caudate	Contralateral	-0.1	1.08	0.34
LIFU-BBBO ∩ Putamen	Ipsilateral	-0.1	1.07	<0.0001
LIFU-BBBO ∩ Putamen	Contralateral	0	1.01	<0.0001
LIFU-BBBO ∩ $(Caudate \cup Putamen)'$	Ipsilateral	0.13	0.78	0.0018
LIFU-BBBO ∩ $(Caudate \cup Putamen)'$	Contralateral	0.16	0.74	0.0018

Variant B

LIFU-BBBO	Ipsilateral	0.04	0.97	<0.0001
LIFU-BBBO	Contralateral	0.20	0.98	<0.0001
LIFU-BBBO ∩ Caudate	Ipsilateral	0.18	0.92	0.16
LIFU-BBBO ∩ Caudate	Contralateral	0.09	1.05	0.16
LIFU-BBBO ∩ Putamen	Ipsilateral	-0.01	1.03	<0.0001
LIFU-BBBO ∩ Putamen	Contralateral	0.18	0.98	<0.0001
LIFU-BBBO ∩ $(Caudate \cup Putamen)'$	Ipsilateral	-0.01	0.86	<0.0001
LIFU-BBBO ∩ $(Caudate \cup Putamen)'$	Contralateral	0.35	0.86	<0.0001

Variant C

LIFU-BBBO	Ipsilateral	0	0.89	<0.0001
LIFU-BBBO	Contralateral	0.12	0.86	<0.0001
LIFU-BBBO ∩ Caudate	Ipsilateral	-0.2	0.94	<0.0001
LIFU-BBBO ∩ Caudate	Contralateral	0.19	0.99	<0.0001
LIFU-BBBO ∩ Putamen	Ipsilateral	0.06	1.01	<0.0001
LIFU-BBBO ∩ Putamen	Contralateral	0.19	0.96	<0.0001
LIFU-BBBO ∩ $(Caudate \cup Putamen)'$	Ipsilateral	-0.02	0.82	<0.0001
LIFU-BBBO ∩ $(Caudate \cup Putamen)'$	Contralateral	0.07	0.78	<0.0001
